# Therapeutic Benefits of Topical Omega‐3 Polyunsaturated Fatty Acids in Skin Diseases and Cosmetics: An Updated Systematic Review

**DOI:** 10.1111/jocd.70341

**Published:** 2025-07-04

**Authors:** Laura Mateu‐Arrom, Ignasi Mora, Leia Garrote

**Affiliations:** ^1^ Department of Dermatology, Hospital de la Santa Creu i Sant Pau, Institut d'Investigació Biomèdica Sant Pau (IIB Sant Pau) Universitat Autònoma de Barcelona (UAB) Barcelona Spain; ^2^ Brudy Technology, S.L. Barcelona Spain; ^3^ Universitat Rovira i Virgili Tarragona Spain; ^4^ Brudy Lab S.L. Barcelona Spain

**Keywords:** atopic dermatitis, DHA, EPA, melanoma, omega‐3 PUFA, psoriasis, skin diseases, topical use, wounds

## Abstract

**Background:**

Oral supplementation with omega‐3 polyunsaturated fatty acids (ω‐3 PUFAs) has shown beneficial effects in some dermatologic diseases (i.e., atopic dermatitis, acne, psoriasis, burns), but the actual effect of local application of ω‐3 PUFAs for improving skin health is still under investigation.

**Aims:**

This paper systematically reviewed the current articles regarding the use of topical ω‐3 PUFAs in the treatment of skin diseases to evaluate its efficacy and safety.

**Methods:**

The review was conducted according to Preferred Reporting Items for Systematic Reviews and Meta‐analysis (PRISMA) recommendations. Studies in which ω‐3 PUFAs were administered as oral treatments, those with a reduced or unknown ω‐3 PUFAs composition, and those using with skin fish grafts were excluded.

**Results:**

In skin models of psoriasis, wounds, dermatitis, and melanoma, topical ω‐3 PUFAs showed an overall beneficial effect associated with the anti‐inflammatory properties of these compounds. Furthermore, none of the studies reported cases of skin irritation, cytotoxicity, or any adverse events.

**Conclusions:**

The increasing number of studies conducted on topical treatments with ω‐3 PUFAs and the existing evidence suggest that ω‐3 PUFAs could be a promising option as an adjuvant or complementary treatment in managing several dermatological conditions. However, there is still a lack of consensus about the optimal dosage, molecules, or delivery methods of ω‐3 PUFAs; consequently, more clinical studies are needed.

## 1 Introduction

**FIGURE 1:**
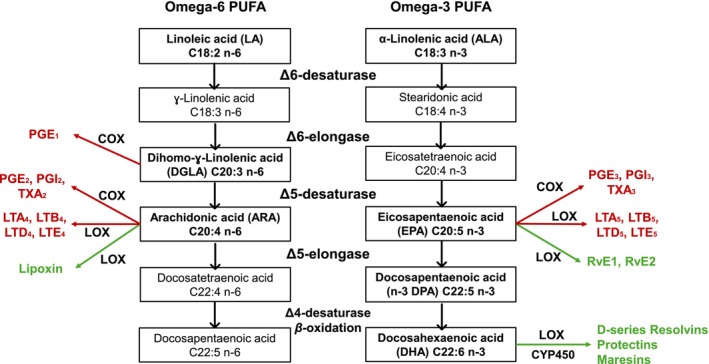
Series of ω‐6 and ω‐3 polyunsaturated fatty acids (PUFAs) and their derived eicosanoids and docosanoids. The inflammatory eicosanoids are highlighted in red, conversely, the inflammation‐resolving eicosanoids and docosanoids are highlighted in green (COX, cyclooxygenase; CYP450, cytochrome 450; LOX, lipoxygenase; LTA, leukotrienes [LTA, LTB LTD, LTE]; PGE, prostaglandin; RvE, resolving E; TXA, thromboxane).

The fatty acids containing ≥ 2 double‐bond desaturations within the acyl chain are considered polyunsaturated. The two main families of polyunsaturated fatty acids (PUFAs) in human health and nutrition are omega‐3 (n‐3, ω‐3) and omega‐6 (n‐6, ω‐6). In both omega series, there are many forms depending on the number of carbons and desaturations, as shown in Figure 1. From the ω‐3 family, α‐linolenic acid (ALA), eicosapentaenoic acid (EPA), and docosahexaenoic acid (DHA) must be highlighted as the most relevant; and from the ω‐6 family, linoleic acid (LA), dihomo‐γ‐linolenic acid (DGLA), and arachidonic acid (AA) must be outlined [1].

Alpha‐linolenic acid (ALA, C18:3 n3) represents the simplest member of the ω‐3 PUFAs family. ALA is the precursor of the long‐chain PUFAs (LC‐PUFAs) EPA (C20:5 n3), docosapentaenoic acid (DPA, C22:5 n3), and DHA (C22:6 n3). These fatty acids are considered essential, and their main source is dietary intake. ALA is present in green leafy vegetables, walnuts, and soybean, whereas EPA and DHA are found in cold water fish, breastmilk, and algae [1]. Oral supplementation with ω‐3 PUFAs has shown beneficial effects in various pathological conditions, especially cardiovascular diseases [2], multiple sclerosis [3] and eye diseases [4]. Since ω‐3 PUFAs are integral components of cell membranes throughout the organism, they exert their functions across various organs and tissues through the modification of cell membrane fluidity, signaling transduction, regulation of gene expression [5], and modulation of the immune response [1].

In the skin, DHA and EPA play an important role in the immune response through several mechanisms [6], including attenuation of the delayed‐type hypersensitivity response through reduction of T‐lymphocyte proliferation and expression of intercellular adhesion molecules [7]; suppressing the expression of the proinflammatory enzyme cyclooxygenase (COX)‐2 and inhibiting activation of the toll‐like receptor (TLR)2 and 4 [8]; and reducing the production of proinflammatory eicosanoids (prostaglandins, thromboxanes, leukotrienes) by increasing the synthesis of inflammation‐resolving lipid mediators (Figure 1) [6]. In fact, as shown in Figure 1 ω‐3 PUFAs are metabolized via the same lipoxygenase and COX pathways as ω‐6 PUFAs; thus, EPA and DHA compete with derivatives of LA (C18:2 n6) such as arachidonic acid (ARA, C20:4 n6) reducing the production of proinflammatory eicosanoids, which are involved in the inflammation and allergic response in cutaneous tissue [9, 10]. In addition, DHA and EPA are ligands of peroxisome proliferator‐activated receptors (PPARs), a transcription factor involved not only in inflammation and immune response, but also in glucose, insulin, and lipid metabolism [6]. As the skin cells are not able to synthesize ω‐3 LC‐PUFAs due to the absence of elongase and desaturase enzyme activity, these LC‐PUFAs should be provided exogenously [1]. Oral supplementation with ω‐3 PUFAs has been associated with beneficial effects in several dermatologic conditions, such as psoriasis [1, 11], atopic dermatitis [12, 13], acne [14, 15], and for improving wound healing in burn patients [16]. Moreover, the use of fish skin grafts has been shown to promote a faster rate of wound healing after burn injury [17], which is in part mediated by the content of ω‐3 PUFA of the grafts.

Based on the pleomorphic properties of ω‐3 PUFAs, there has been a growing interest in developing topical formulations of ω‐3 PUFAs for skin health. In 2018, Huang et al. [10] published a systematic review reporting beneficial effects of the cutaneous application of fatty acids derived from fish oil, especially ALA, LA, DHA, and EPA, in cell‐based, animal‐based, and clinical models. The authors concluded that although these products have demonstrated efficacy in improving skin barrier function, inhibiting UV‐induced inflammation and hyperpigmentation, attenuating dry skin and pruritus elicited by dermatitis, and accelerating wound healing, data from clinical trials for skin application of ω‐3 PUFAs are still limited [10]. This systematic review aimed to update information and synthesize the recent evidence on the efficacy and safety of the topical use of ω‐3 PUFAs in the treatment of skin conditions. The present findings will help dermatologists in future investigations and in clinical decision making by providing the latest data insights of the indications and beneficial impact of the topical application of ω‐3 PUFAs.

## 2 Materials and Methods

### 2.1 Search Strategy

This systematic review followed the Preferred Reporting Items for Systematic Reviews and Meta‐analyses (PRISMA) statement [18]. Articles of topical ω‐3 PUFAs were searched in PubMed and Scopus databases by one of the authors (I.M.). The search strategy was based on the Medical Subject Headings (MeSH) terms and relevant free text items present in the title, abstract, or keywords. The following search terms were used: (omega‐3) OR (fish oil) OR (PUFA) OR (polyunsaturated fatty acids) AND (skin) OR (cosmetic) OR (topical). The results were filtered to include only full‐text research articles published in English since 2018. This timeframe was chosen as a previous literature review on a similar topic had been published including studies conducted until 2018 [10]. The search strategy was adapted for each database and was performed in September 2024.

### 2.2 Inclusion and Exclusion Criteria

Only studies describing results of topical treatments with ω‐3 PUFAs in mammalian skin in vitro models, mammalian skin animal models, or humans were considered for inclusion. Exclusion criteria were as follows: articles published in any language other than English; studies focused on non‐cutaneous diseases; studies assessing non‐topical application of ω‐3 PUFAs (e.g., oral supplementation); studies in which ω‐3 PUFAs content was less than 10% of the investigational product or was not reported; studies of fish‐skin grafts; review articles, editorials, conference presentations, opinion/comments, abstracts, and cross‐sectional studies; and studies in which the methodology was not fully reported. Two authors (L.G. and L.M.‐A.) conducted a full‐text screening of all eligible articles to ensure that all inclusion criteria and no exclusion criteria were met.

### 2.3 Data Extraction

One researcher (I.M.) developed a data extraction form, which was completed for all selected articles. The form included key study details such as authors, publication year, treatment protocol, patients or experimental models, and main results (Tables S1–S3). The entire team (L.M.‐A., I.M. and L.G.) validated the data entry.

### 2.4 Quality Assessment

Since the reports of the current review included in vitro, preclinical, and human studies, the risk of bias of the included studies was not scored. The methodological quality of the records was independently assessed by two investigators (I.M. and L.M.‐A.) and agreed upon by the entire team.

### 2.5 Statistical Analysis

Descriptive statistics based on results reported in the studies included in the review are here presented.

## 3 Results

**FIGURE 2:**
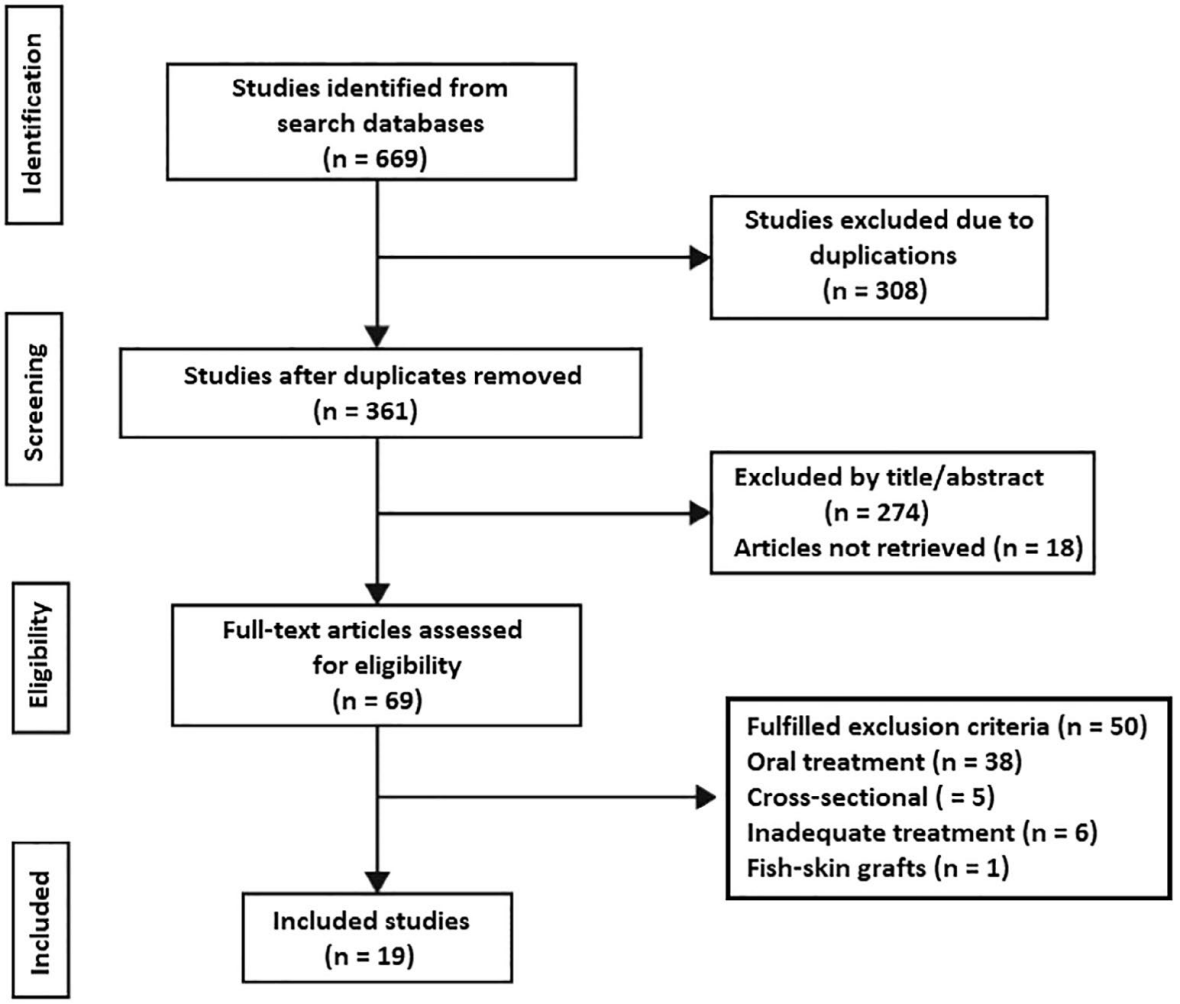
Flow chart of the studies included in the review.

The initial literature search retrieved 669 articles, which were then narrowed down to 69 records by removing duplicates, screening titles and abstracts, and records that could not be retrieved. Of these 69 articles, 50 were finally excluded because of treatment with ω‐3 PUFAs by the oral route, cross‐sectional design, ω‐3 PUFAs content < 10% of the investigational product or undefined dose, and fish‐skin grafts. Finally, 19 articles were included in the review (Figure 2).

### 3.1 General Characteristics of Studies

A total of 19 studies were included in the review, and most of them (*n* = 13) were performed in mammalian skin in vitro models, followed by preclinical animal models (*n* = 7) and studies in humans (*n* = 3). Some studies included both in vitro and animal models. In relation to in vitro cell line models, all of which but one were carried out in cells extracted from human patients or healthy donors. Cell lines included keratinocytes, dermal fibroblasts, or immune cells. One study was performed in melanoma cells from patients and from mice, whereas keratinocytes from mice were used in another study. Disease conditions were psoriasis in five studies, skin healing in 3, inflammation or stress response in 2, skin hydration in 2, and melanoma in one study. DHA, EPA, and ALA were the ω‐3 PUFAs evaluated. Animal models in rodents were used in seven studies assessing the effect of DHA, ALA, or EPA. The conditions evaluated included skin healing, stress response, diabetes ulcers, psoriasis, and inflammation, and cancer prevention. Only three studies were performed in humans; 2 of them analyzed the outcomes of ALA for skin hydration, and 1 assessed the hydration and anti‐inflammatory properties of DHA. The details of these studies are shown in the Tables S1–S3.

Following the framework of the previous review [10], the present findings were described according to the dermatological condition and results reported according to the use of in vitro mammalian skin models, animal models, or human beings.

### 3.2 Topical Use of ω‐3 PUFAs in Skin Models of Psoriasis

Psoriasis is a chronic inflammatory skin disease which usually presents as elevated, well‐defined, reddish oval patches with silvery scales, resulting from abnormal keratinocyte proliferation and differentiation, accompanied by immune cell activation and infiltration [19]. It affects between 0.11% and 1.58% of people worldwide, with variation based on geographical region [20], and it is associated with a significant psychological burden and a high impairment of health‐related quality of life [21]. Although the pathogenesis of psoriasis is not fully understood, it is now recognized as an immune‐mediated disorder with a complex interplay between the innate and adaptive immune systems, influenced by either genetic or environmental risk factors [22]. Moreover, it has been shown that psoriatic skin has a specific profile of bioactive lipid mediators, with higher amounts of ω‐6 PUFAs (LA and ARA) and their derivatives, such as leukotriene B4 (LTB4), 12‐hydroperoxyeicosatetraenoic acid (12‐HPETE), and prostaglandin (PG) E_2_, compared to healthy skin [23].

The Table 1 shows the results obtained of the use of ω‐3 PUFAs in skin models of psoriasis. Most of the evidence was based on data from in vitro studies. The group of Morin and Simard has conducted several studies assessing the effects of ω‐3 PUFA supplementation in three‐dimensional healthy and psoriatic skin models created by tissue engineering [24, 25, 27, 28, 29]. To assess the effects of ALA on the proliferation and differentiation of psoriatic keratinocytes, the results of 10 μM of ALA supplemented medium in both psoriatic and healthy skin models were compared to a non‐supplemented medium [25]. An increase in EPA‐derived lipid mediators and a decrease in ω‐6 PUFA‐derived lipid mediators after ALA treatment were observed, probably through replacing ARA in the molecular pathways [25]. As a result, a reduction in keratinocyte proliferation and an improved epidermal differentiation, with an increased expression of filaggrin and loricrin, were found. Levels of filaggrin were also increased in healthy skin models after ALA supplementation, indicating that it could also have a beneficial effect on healthy skin [25]. Similar results were found when the culture medium was supplemented with 10 μM of DHA [24]. Psoriatic DHA‐supplemented models showed a thinner epidermis, more likely to the histological appearance of healthy skin, and an enhanced filaggrin and keratin10 expression, indicating a better epidermal differentiation and decreased levels of the proinflammatory biomarkers COX‐2 and tumor necrosis factor (TNF) alpha (TNFα) [24].

**TABLE 1:** Studies assessing the effect of topical ω‐3 PUFAs treatment on psoriatic skin models.

First author, year [reference]	Treatment	Experimental model	Main outcomes
Morin, 2021 [24]	Culture medium with 10 μM of DHA	A 3D tissue culture with fibroblasts and keratinocytes from patients	Decreased PGE_2_ and 12‐HETE Rebalance expression of PPARs Decreased TNFα Overall, attenuates psoriatic characteristics
Simard, 2021 [25]	Culture medium with 10 μM of ALA	A 3D tissue culture with fibroblasts and keratinocytes from patients	Increased EPA and decreased ω‐6 derivatives Decreased psoriatic phenotype by normalizing keratinocyte proliferation and differentiation
Mittal, 2021 [26]	Doses of 500 mg nanoemulsion containing fish oil or linseed oil with tacrolimus (0.1% w/v), applied to the shaved skin of the mice	Adult albino mice (imiquimod‐induced psoriasis‐like inflammation model)	Decreased PASI score Reduced inflammatory cytokines (TNFα and IL‐6) in the skin Increased drug skin permeation of tacrolimus
Morin, 2022 [27]	Culture medium supplemented with ALA	A 3D tissue culture with fibroblasts, keratinocyte and activated T cells from patients	Reduced infiltration of T cells into epidermis. Decreased inflammatory cytokines and chemokines (CXCL, IL‐6 and IL‐8)
Morin, 2023 [28]	Culture medium with EPA 10 μM	A 3D tissue culture with fibroblasts, keratinocyte, and polarized T cells from patients	Increased ω‐3 PUFA in membrane phospholipids. Regulated lipid composition Increased PGE_3_, 12‐HEPE and EPEA levels Promoted homeostasis
Morin, 2023 [29]	Culture medium with 10 μM EPA	A 3D tissue culture with fibroblasts, keratinocyte, and polarized T cells from patients	Normalized proliferation of psoriatic keratinocytes Modified NFκB pathway Reduced IL‐17A‐positive cells

Abbreviations: 12‐HEPE, 12‐hydroxyeicosapentaenoic acid; 12‐HETE, 12‐hydroxyeicosatetraenoic acid; ALA, α‐linolenic acid; CXCL, chemokine (C‐X‐C motif) ligand; DHA, docosahexaenoic acid; EPA, eicosapentaenoic acid; EPEA, *N*‐eicosapentaenoylethanolamine; IL, interleukin; NFκB, nuclear factor kappa light chain enhancer of activated B cells; PASI, Psoriasis Area and Severity Index; PGE_2_, prostaglandin E2; PPARs, perosyxomel proliferator activated receptors; TNFα, tumor necrosis factor alpha.

The same authors also investigated the effect of supplementation with ω‐3 PUFAs on immune cells by the addition of T cells in the healthy and psoriatic skin models [27, 29]. In one study, T cells isolated from blood samples of healthy donors and then activated were added to psoriatic and healthy skin models and supplemented with 10 μM of ALA. It was found that ALA reduced the number of T cells infiltrating the psoriatic skin model, especially the epidermis [27]. The supplementation with ALA also caused a significant decrease of inflammatory cytokines, such as interleukins (IL) IL8, IL6, TNFα or gamma interferon (IFN‐γ), and a tendency to reduce IL‐17A [27] secretion. In fact, in the skin models mixed with T cells, the addition of 10 μM of EPA resulted in an upregulation of the ω‐3 PUFA lipid mediators and a decrease in the ω‐6 PUFA derivatives [28], producing a lower proportion of IL‐17A‐producing T cells and an increase in the proportion of T cells expressing FOXP3, which is a marker of regulatory T cells [29].

In animal models, Mittal et al. [26] used an imiquimod‐induced psoriasis‐like inflammation mice model to assess the efficacy of a nanoemulsion gel of tacrolimus prepared with fish oil, rich in DHA and EPA, and linseed oil, rich in ALA. After obtaining an optimal formulation, the drug permeation in the skin between the nanogel formulation and a marketed tacrolimus product using confocal microscopy was compared. It was found that there was deeper permeation and more deposition of tacrolimus in the epidermal layers of the skin in favor of the nanoemulsion formulation. Also, in mice treated with the nanoemulsion gel, there was a significantly higher reduction of the Psoriasis Area and Severity Index (PASI) score without signs of skin irritation in any group, whereas the levels of IL‐6 and TNFα showed a maximum reduction in this treatment group [26].

### 3.3 Topical Use of ω‐3 PUFAs in Wound Healing

Wound healing is a complex and dynamic process with several overlapping phases. In the skin, there is an initial phase of inflammation and recruitment of cells to combat potential infections, along with migration and proliferation of fibroblasts and keratinocytes from adjacent sites, followed by angiogenesis, synthesis of extracellular matrix with accumulation of collagen fibers that ends with complete re‐epithelialization and restoration of tissue integrity. Since the inflammatory reaction and its resolution play a crucial role for the initiation of wound healing, several agents including PUFAs have been advocated due to their immunomodulatory effects [30, 31]. Acellular fish‐skin grafts which are rich in EPA and DHA and promote inflammatory‐resolving lipid mediators [32] have been tested in animal and human wounds, showing positive results in terms of shortening time of wound healing, less pain, or better aesthetic and functional outcomes compared to standard dressings [18]. However, the beneficial effects of such grafts could be mediated by other components of the fish‐skin such as their matrix, their surface structure and texture, or the presence of growth factors [33].

Regarding the latest evidence on topical ω‐3 PUFA treatment in wound healing, three in vitro studies and two preclinical studies were reviewed (Table 2). Severing et al. [33] assessed the effects of 150 μM of EPA or 150 μM of DHA on metabolic activity, cell proliferation, and migration of human keratinocytes and fibroblasts cultured with human chronic wound fluid to simulate the microenvironment of a chronic wound. Although the supplementation of ω‐3 PUFAs showed an initial increase in metabolic activity, which was further counteracted by the effects of the chronic wound fluid, a significant effect on cell proliferation or wound closure was not observed. However, human keratinocytes and fibroblasts exhibited a significant increase in metabolic activity when treated with DHA and EPA [33].

**TABLE 2:** Studies assessing the effect of topical ω‐3 PUFAs treatment on would healing.

First author, year [reference]	Treatment	Experimental model	Main outcomes
Ontoria‐Oviedo, 2022 [34]	LIPINOVA oil with standardized levels of 17‐HDHA, 18‐HEPE, 14‐HDHA, EPA, and DHA. Dosage of 50 μM and 250 nM in cells. Daily dosage of 50 ng in mice	– Human dermal fibroblasts and keratinocytes from patients. Monocytes (CD14+ cells) isolated from buffy coats of healthy donors – Adult type 2 diabetes (*db*/*db*) mice	– Decreased expression of IL‐1β and CXCL10 genes, and lower amount of CXCL10 in macrophages. Promotion of in vitro wound closure – Improved wound healing closure – Promoted thicker parakeratotic stratum corneum, mature dense connective tissue and induces angiogenesis – Macrophage phenotype switches from pro‐inflammatory to pro‐resolving
Severing, 2022 [33]	Supplementation with 150 μM DHA or EPA	HaCaT human keratinocytes CRL‐2522 newborn foreskin fibroblasts	– Increased the metabolic activity of keratinocytes and fibroblasts – No significant positive effect on skin cell proliferation and wound closure
Torrissen, 2023 [35]	Culture medium with DHA or ω‐3 VLC‐PUFA concentrate at 3 μM	PCS‐201‐012 human dermal fibroblasts	Modulated gene expression and produced greater rate of fibroblast migration, favoring in‐vitro scratch healing
Mititelu, 2023 [36]	Emulsion‐gel of stingray liver oil; approximately 0.25 g of gel per rat's paw (2.45 mg of ω‐3 PUFAs)	Male Wistar rats, paw edema caused with intra‐plantar injection of kaolin and dextran solution	The treatment produced completely wound regeneration after 12 days and fast resolution of inflammatory edema after induction
El‐Sheekh, 2024 [30]	Topical cream rich in PUFA extracted from microalga *Parachlorella kessleri* (ALA 22.8%)	Male Swiss albino mice (CD‐1 strain) with two bilateral wounds or full‐thickness burns on the back	Significant effect on the reduction of excisional wounds and burns Histopathological analysis showed an improvement of angiogenesis, collagen fiber formation, and epidermis creation

Abbreviations: ALA, α‐linolenic acid; CXCL10, chemokine (C‐X‐C motif) ligand 10; DHA, docosahexaenoic acid; EPA, eicosapentaenoic acid; HDHA, hydroxy docosahexaenoic acid; HEPE, hydroxyeicosapentaenoic acid; IL, interleukin; VLC‐PUFA, very‐long‐chain polyunsaturated fatty acids.

The effect of very‐long‐chain PUFAs (VLC‐PUFAs) on the migration and proliferation properties of human dermal fibroblasts and Atlantic salmon keratocytes was evaluated by Torrissen et al. [35]. In this study, 10–20 μM of a 93% concentrate of VLC‐PUFAs was added to the salmon keratocytes, and 3 μM of VLC‐PUFA and DHA to the dermal fibroblast media. Cell migration of keratocytes from fish scales was significantly higher in cultures enriched with VLC‐PUFAs as compared with controls. Moreover, after creating a scratch in the center of the fibroblast culture (scratch‐wound assay), a 10% difference in reduction of the scratch size in favor of the VLC‐PUFA supplemented group compared to DHA and control groups was found. The gene expression profile in cultured dermal fibroblasts after supplementation was also analyzed, and significant differences in gene expression between groups, affecting genes related to lipid metabolism, inflammation, cell proliferation, and cell growth were observed [35].

The topical effect of a commercially available formulation (LIPINOVA) enriched with anchovy and sardine oils in the healing process of diabetic ulcers in in vitro and animal models was assessed [34]. The formula contained standardized levels of 17‐HDHA (hydroxy‐4Z,7Z,10Z,13Z,15E,19Z‐docosahexaenoic acid), 18‐HEPE (hydroxy‐5Z,8Z,11Z,14Z,16E‐eicosapentaenoic acid), 14‐HDHA, EPA, and DHA. The product was demonstrated to be absorbed by keratinocytes, fibroblasts, human macrophages, and human umbilical vein endothelial cells. The cytotoxicity of different concentrations of the product on cultured keratinocytes and fibroblasts showed that concentrations lower than 250 μM for keratinocytes or 125 μM for fibroblasts did not affect the viability of the cells. The product also demonstrated the ability to induce migration of keratinocytes and fibroblasts in an in vitro scratch‐wound assay [34]. Additionally, the formula suppressed the expression of type 1‐macrophage markers and promoted a higher expression of type 2‐macrophage markers, associated with inflammation resolution and tissue repair processes. Finally, the same product was tested in wounds of 10 diabetic mice randomized to receive the formulation or saline. Wounds were completely closed, with increased blood vessel density and type‐2 macrophage polarization in the treated mice but not in controls [34].

In another preclinical study, Mititelu et al. [36] evaluated the wound healing and anti‐inflammatory effect of a dermo‐cosmetic preparation (7 g of stingray liver oil containing 14% of ω‐3 PUFAs per 100 g of the product) in laboratory rats. After 8 days of treatment, a cure rate of 79% was recorded in animals treated with the prepared ointment as compared with 65% in those treated with a reference cicatrizing ointment and 44% in untreated animals. Moreover, inflammatory edema was induced by injection of a 10% kaolin suspension and dextran gel in 60 rats. Then, stingray liver oil ointment or a diclofenac gel was applied and compared to the control (left untreated). The results showed a significant reduction of edema in both treatment groups as compared with the untreated group [36].

At last, El‐Sheekh et al. [30] cultivated the microalga *Parachlorella kessleri* in a glycine culture and extracted the oil with a 22.8% content of ALA. After assessing its antioxidant properties, the *P. kessleri* oil was tested and compared to placebo in 30 mice in which excisional wounds or burns were created. There was a significant effect on the wound area reduction compared to untreated wounds, with closure rates of 97% in excisional wounds and 98% in burns. These results were also correlated with histopathological changes, with more layers of epithelial cells and less intense inflammatory infiltrate in treated wounds.

### 3.4 Topical Use of ω‐3 PUFAs in Skin Inflammation and Irritation

Damage of the stratum corneum by direct action of chemical, physical, or mechanical agents may cause skin irritation [37]. Inflammation is the reactive response to tissue damage aimed to eliminate harmful agents and promote tissue regeneration [38]. Inappropriate or prolonged skin irritation and inflammatory response are common complaints of skin diseases [38, 39].

One in vitro study and three studies in animal models were included in the review, the characteristics of which are shown in Table 3. In human keratinocyte cell lines treated with 10 and 30 μM of DHA loaded in a resveratrol‐based solid lipid nanoparticle formulation, the influence on the production of proinflammatory cytokines (IL‐1β, IL‐6, and MCP‐1) and reactive oxygen species (ROS) formation induced by TNFα or the surfactant sodium dodecyl sulfate (SDS) was evaluated [38]. The study formulation reduced SDS‐induced cytotoxicity and the IL‐1β, IL‐6, and MCP‐1 production in the two keratinocyte cell lines, as well as the NLRP3 inflammasome activation and ROS production caused by SDS and TNFα [38].

**TABLE 3:** Studies assessing the effect of topical ω‐3 PUFAs treatment on skin irritation and inflammation.

First author, year [reference]	Treatment	Experimental model	Main outcomes
Serini, 2019 [38]	Resveratrol‐stearate nanoparticles loaded with DHA at 10–30 μM	HaCaT and NCTC‐2544 human immortalized keratinocytes	Inhibited production of IL‐1β, IL‐6, and MCP‐1, as well as the NLRP3 inflammasome and ROS after induction with SDS and TNFα
Jamil, 2020 [40]	DHA (10 μmol), 17‐oxo‐DHA (20 nmol), and 17‐OH‐DHA (20 nmol) dissolved in 200 μL of acetone. Compounds applied onto the dorsal skin of mice for 2.5 or 5 h	Female HR‐1, hairless mice	Disrupted proteasome‐mediated degradation of Nrf2 by Keap1 binding Elevated nuclear localization of Nrf2 and expression of heme oxygenase‐1 (HO‐1) Enhanced antioxidant response
Ames, 2020 [41]	Dosage of 0.25, 0.5, 1, 2, and 4 mg of DHA (from fish oil) per ear	Healthy Wistar rats	Inhibition of MPO content, indirect marker of neutrophil recruitment in inflamed tissue. No reduction of ear edema intensity
Kim, 2023 [42]	17‐Oxo‐DHA (20 nmol) dissolved in 200 μL of acetone and applied in the dorsal skin of mice 30 min prior to UVB exposure	Female SKH1‐Hr^hr^ mice (hairless) irradiated with UVB (180 mJ/cm^2^)	Reduced levels of 4‐hydroxynonenal (HNE)‐modified protein, MDA, and 8‐Oxo‐dG. Increased activation of Nrf2 Reduced TNFα and IL‐6 production and expression

Abbreviations: DHA, docosahexaenoic acid; IL, interleukin; MCP‐1, monocyte chemoattractant protein‐1; MDA, malondialdehyde; MPO, myeloperoxidase; NLRP3, nucleotide‐binding domain, leucine‐rich‐containing family, pyrin domain‐containing‐3; Nrf2, nuclear factor erythroid 2‐related factor 2; Oxo‐dG, 8‐oxo‐2′‐deoxyguanosine; ROS, reactive oxygen species; SDS, sodium dodecyl sulfate; TNFα, tumor necrosis factor alpha.

Associated with the inflammatory‐resolving species derived from DHA, Jamil et al. [40] investigated the antioxidant effects of 17‐oxo‐DHA, a DHA metabolite generated by COX‐2 and dehydrogenases in macrophages, and compared its effect to the non‐electrophilic metabolic precursor 17‐hydroxy‐DHA (17‐OH‐DHA), with Nrf2‐mediated expression of heme oxygenase‐1 (HO‐1) as the main study variable. Briefly, under non‐stressed conditions, Nrf2 forms a complex with the protein Keap1. Upon an oxidative stimulus, Keap1 degradation occurs, releasing Nrf2, which then activates the transcription of genes involved in antioxidant pathways, such as HO‐1. Mouse epidermal cells treated with 5 μM of 17‐oxo‐DHA or 5 μM of DHA were used. It was found that 17‐oxo‐DHA increased the level of Nrf2 located in the nucleus and induced more Keap1 degradation and HO‐1 protein expression than DHA. Also, either DHA (10 μmol), 17‐oxo‐DHA (20 nmol), or 17‐OH‐DHA (20 nmol) dissolved in 200 μL of acetone were topically applied to the dorsal skin of mice. It has been shown that DHA, and to a greater extent 17‐oxo‐DHA, elevated the expression of Nrf2 and its target proteins while decreasing the expression of Keap1 [40].

In a mice model, Kim et al. [42] investigated the effects of 17‐oxo‐DHA on UVB‐induced oxidative stress, inflammation, and skin carcinogenesis. Topical use of 17‐oxo‐DHA reduced UVB‐induced dermatitis and oxidative stress, as shown by decreased markers of tissue and DNA damage. It was also observed upregulation of Nrf2 and its target proteins such as HO‐1, decreased expression of proinflammatory cytokines TNFα and IL‐6, and reduced phosphorylation of STAT3, a proinflammatory transcription factor that enhances cell growth through c‐Myc and cyclin D1, promoting inflammation and cancer. Moreover, long‐term application of 17‐oxo‐DHA before UVB exposure led to a significant reduction in the number of papillomas and tumors in mouse skin compared to control mice [42].

Furthermore, the effect of fish oil preparation with different amounts of DHA (0.25, 0.5, 1, 2, and 4 mg) in a phenol‐induced ear edema in a mice model was assessed [41]. The product showed percutaneous penetration into the ear tissue, as measured by photoacoustic spectroscopy. Although treatment did not reduce edema, it was associated with a decrease in myeloperoxidase content, which is considered a marker of neutrophil recruitment during inflammation [41].

### 3.5 Topical Use of ω‐3 PUFAs in Skin Cancer

Skin cancers, either melanoma or nonmelanoma, have been associated with oxidative stress and a chronic inflammatory process of the skin [43], with UV radiation being a major risk factor followed by immunosuppression. Omega‐3 PUFAs have shown benefit in cell growth and carcinogenesis, in both in vitro and in vivo animal models, and as dietary supplements in humans [10].

In human and mice metastatic melanoma cell lines, the effect of 10–30 μmol/L of DHA or EPA on modulating the DNA repair activity and enhancing the response to cisplatin was evaluated [44]. It was found that the combination of DHA and cisplatin induced a 40.2% human cell growth inhibition, whereas cisplatin alone inhibited cell growth by 13.5%. The combination of cisplatin and DHA also proved to inhibit melanoma cell migration and to reduce the expression of the DNA‐repairing enzyme ERCC1, involved in the resistance of melanoma cells to the action of cisplatin [44]. Also, a reduction of phosphorylated ERK1/2 and an increase in the expression of DUSP6, both related to the cisplatin‐induced ERCC1 expression, were also observed in the treatment group. These findings suggested that DHA and EPA could enhance the responsiveness of melanoma cells to cisplatin's antineoplastic effects by altering the ERCC1/ERK1/2/DUSP6 DNA repair pathway [44].

In another study in which the development of tumors in mice exposed to the UVB was evaluated [42], treatment with topical 17‐oxo‐DHA prior to irradiation was associated with a lower number of papillomas and tumors than those found in vehicle‐treated animals, with a reduction of 42.4% in tumor burden, particularly of large tumors. Decreased levels of STAT3 phosphorylation and subsequent expression of the oncoprotein c‐Myc, as well as suppressed angiogenesis in skin tumor tissues of mice treated with 17‐oxo‐DHA as compared to controls, were also observed [42]. A summary of these studies is shown in Table 4.

**TABLE 4:** Studies assessing the effect of topical ω‐3 PUFAs treatment on skin cancer models.

First author, year [reference]	Treatment	Experimental model	Main outcomes
Ottes‐Vasconcelos, 2019 [44]	DHA and EPA at 10–30 μM in culture medium	B16F10 murine melanoma cells. WM266‐4 human melanoma cells	Sensitized tumoral cells to cisplatin through DUSP6/p‐ERK/ERCC1 repair pathways after cisplatin‐induced inhibition of cell growth and migration
Kim, 2023 [42]	17‐oxo‐DHA (20 nmol) dissolved in 200 μL of acetone and applied in the dorsal skin of mice 30 min prior to UVB exposure	Female SKH1‐Hr^hr^ mice (hairless) irradiated with UVB (180 mJ/cm^2^)	Reduced STAT3 phosphorylation (oncoprotein activator) induced by UVB. Protected against mouse skin tumor development

Abbreviations: DHA, docosahexaenoic acid; EPA, eicosapentaenoic acid; STAT3, signal transducer and activator of transcription 3; UVB, ultraviolet B.

### 3.6 Topical Use of ω‐PUFAs for Skin Hydration

Skin dryness is usually caused by alterations in the composition or structure of the stratum corneum, which leads to an increase in water loss and impairment of the barrier function facilitating skin inflammation [45]. Thus, substances with the capability of maintaining the integrity of the stratum corneum could be beneficial for ensuring proper skin hydration.

The safety and efficacy of *Plukenetia volubilis* L. (sacha inchi) oil (42.2% of ALA and a 39.5% of LA) for maintaining skin hydration were evaluated in cultures of human skin cells obtained from 6 (3 control and 3 treated) human full‐thickness skin biopsies, and in 15 volunteers who were randomized to apply sacha inchi oil or olive oil (benchmark) on the left or right leg, respectively [45]. As the sacha inchi oil did not cause secretion of TNFα and IL‐1α, or disruption of keratin 1 (a marker for terminal differentiation of epidermal cells) in the stratum corneum, it was concluded that the oil was safe, without irritant effects for the skin. After 16 days of treatment, the patients with sacha inchi oil showed increased values of moisture content (measured with a corneometer), and improvement of skin dryness appearance comparable to the group treated with olive oil [45].

The properties of HYVIA, a chia ( *Salvia hispanica* L.) seed extract with up to 31.22 μg/mL of ALA and 8.86 μg/mL of LA, were evaluated in cell cultures and in healthy volunteers [46]. The product inhibited protein phosphatase 2A (PP2A) demethylation, maintaining the active form of this enzyme, which is involved in the degradation of filaggrin. HYVIA also showed an increase in gene expression activity of aquaporin‐3 (AQP3) and hyaluronic acid synthase 2 (HAS2), markers of skin hydration in human epidermal keratinocyte cultures. In the single‐blind clinical trial conducted with 16 healthy volunteers, skin hydration and transepidermal water loss were measured after applying a 0.1% HYVIA cream or a vehicle cream to the leg. At 24 h, HYVIA‐treated skin was 58% more hydrated than vehicle‐treated skin, whereas significantly lower transepidermal water loss values were detected in HYVIA‐treated skin with respect to untreated skin areas [46].

Finally, a prospective multicenter masked trial was performed with 60 individuals (30 contact lens users and 30 noncontact lens users), testing a moisturizing gel‐cream containing Tridocosahexanoina‐AOX, a DHA concentrated triglyceride (0.7%), sodium hyaluronate (0.2%) and *Aloe vera* (3%) [47]. The gel was applied on the right upper and inner eyelids while the left eye was used as a control. After 2 weeks of treatment, it was found that there was a significant improvement in the Schirmer's test and fluorescein break‐up time in the treated eye with respect to the control eye, and a trend toward a diminished level of proinflammatory cytokines in the tear samples of treated eyes. The vast majority of patients reported that the treated eye had improved skin brightness, softness, and elasticity, with fewer wrinkles, whereas none of them presented adverse events [47]. A summary of these studies is shown in Table 5.

**TABLE 5:** Studies assessing the effect of topical ω‐3 PUFAs treatment on skin hydration.

First author, year [reference]	Treatment	Experimental model	Main outcomes
Soimee, 2020 [45]	– Incubation of cells for 24 h with 20 μL of sacha inchi oil – Application in left or right lower leg of 0.5 mL of sacha inchi oil twice a day for 14 consecutive days	– Skin tissues specimens from females of 50–65 years old after surgery – 13 healthy females aged between 20 and 60 years	– Reduced secretion of TNFα and IL‐1. Reduced disruption of keratin 1 integrity in the stratum corneum layer – Beneficial effect on dry skin. Moisturizing effect comparable to that of olive oil. Proved safety of the treatment
Huber, 2020 [46]	– Emulsion formulated with 0.1% HYVIA, chia seed extract – Dosage of 50 μL HYVIA applied in two 25 cm^2^ areas of the lower legs	– Human epidermal keratinocytes (NHEKs) from neonatal donors – 16 subjects, 5 males–11 females, aged between 18 and 60 years	– PPA2 activation involved in filaggrin degradation. Improved expression of AQP3 and HAS2 skin hydration markers – Skin hydration improved by 16% and 58% over vehicle at 2 and 24 h post‐treatment, respectively. Significantly lower transepidermal water loss (TWEL) values compared to untreated skin
Pinazo‐Duran, 2021 [47]	Approximately 0.2 mL of gel‐cream containing 0.7% of DHA in a triglyceride form in the skin of the right closed eyelids	68 participants aged 22–60 years	No reduction of inflammatory cytokine/chemokine in tears Improvement in skin brightness, softness, elasticity, and hydration, with no adverse effects

Abbreviations: AQP3, aquaporin 3; DHA, docosahexaenoic acid; HAS2, hyaluronan synthase 2; IL, interleukin; PPA2, inorganic pyrophosphatase 2; TNFα, tumor necrosis factor alpha.

## 4 Discussion

The present review updates information reported by Huang et al. [10] in 2018 focused on the application of fish oil for cosmetic and therapeutic approaches, the primary effect of which is attributed to ω‐3 PUFAs. There is limited evidence of the benefits of the topical use of ω‐3 PUFAs in dermatological conditions, and a comprehensive search of the literature from 2018 provided only 19 studies. Overall, topical ω‐3 PUFAs showed a positive effect for improving skin health in different conditions, such as psoriatic models, irritation and inflammation, wound healing, and hydration, highlighting the anti‐inflammatory effect shown in almost all studies. It should be noted that the topical use of agents of the ω‐3 PUFA family, particularly DHA and EPA, was well tolerated and safe, and none of the studies reported adverse events, cytotoxicity, or local skin reactions.

The present findings consistently showed that topical use of ω‐3 PUFAs reduces inflammation and improves keratinocyte proliferation and differentiation in psoriatic models, reducing PASI scores in animal studies and in healthy volunteers. Moreover, they promoted proliferation and migration of human keratinocytes and fibroblasts, prompting wound closure in in vitro and preclinical studies. However, in an experiment of human chronic wound fluid (CWF) for simulation of the microenvironment of a chronic wound, the addition of DHA or EPA showed no relevant benefit for skin cells challenged with CWF [33]. On the other hand, ω‐3 PUFAs successfully modulated the production of proinflammatory cytokines in models of dermatitis and skin irritation, potentially playing an important role in skin cancer prevention. Furthermore, ω‐3 PUFAs slightly improved skin hydration by diminishing transepidermal water loss, which is a key point to prevent the development of dermatitis.

**FIGURE 3:**
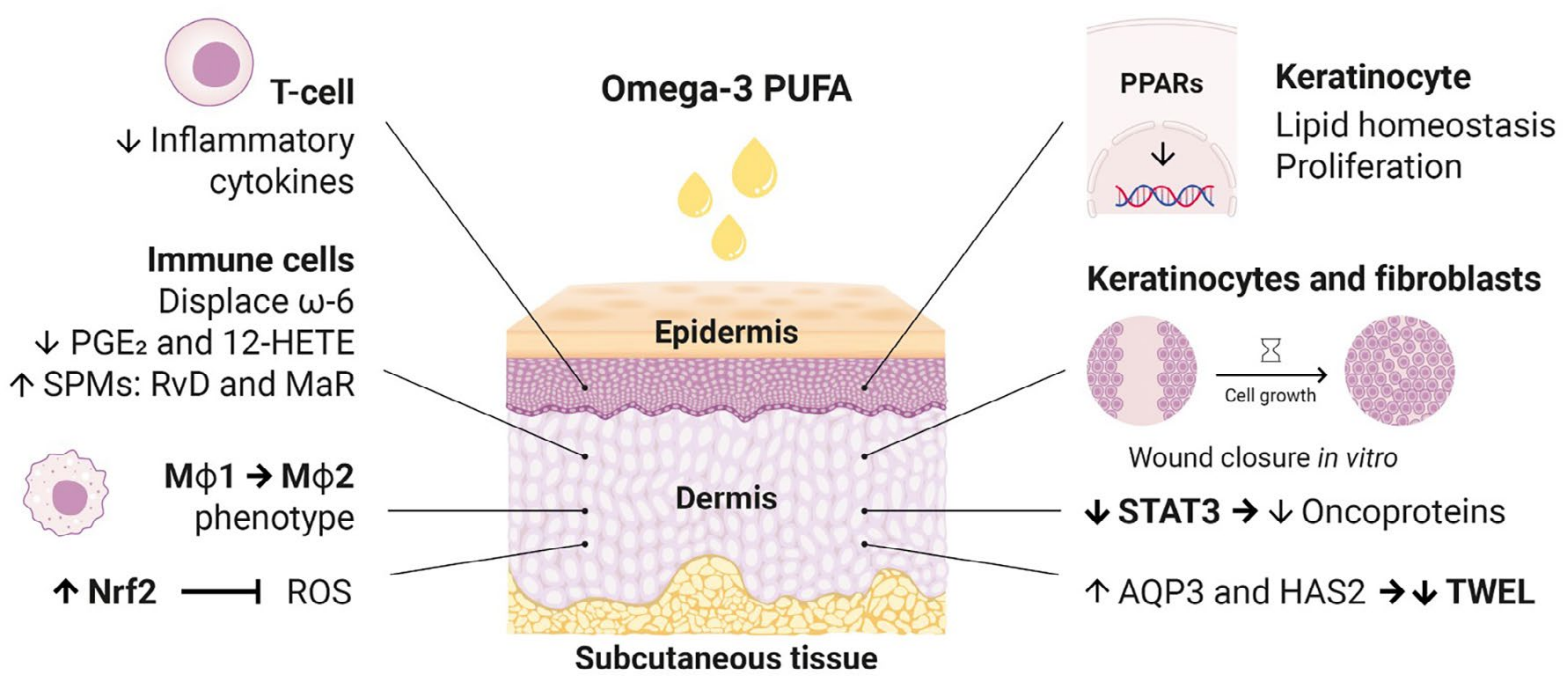
Summary of the beneficial effects of the topical treatment with ω‐3 PUFAs described in the review for different skin conditions (ω‐6, omega‐6 PUFA; 12‐HETE, 12‐hydroxyeicosatetraenoic acid; AQP3, aquaporin 3; arrows up, increase; down arrows, decrease; HAS2, hyaluronan synthase 2; Mϕ, macrophague; Nrf2, nuclear factor erythroid 2‐related factor 2; PGE, prostaglandin E‐series; PPARs, peroxisome proliferator–activated receptors; ROS, reactive oxygen species; STAT3, signal transducer and activator of transcription 3; TWEL, transepidermal water loss).

A summary of the actions of ω‐3 PUFAs in the epidermis and dermis layers is shown in Figure 3. At the level of the epidermis, ω‐3 PUFAs contribute to lipid homeostasis, proliferation of keratinocytes, modulation of peroxisome proliferator‐activated receptors (PPARs), which are essential for maintaining skin barrier permeability and regulation of skin inflammation, and a decrease of inflammatory cytokines. In the dermis, ω‐3 PUFAs exhibit multiple effects on keratinocytes, fibroblasts, immune cells, and macrophages, influencing signaling pathways involving prostaglandin E compounds, reactive oxygen species, nuclear factor erythroid 2‐related factor, signal transducer and activator of transcription 3, aquaporin 3, and hyaluronan synthase 2.

In this review, most evidence regarding the effects of topical ω‐3 PUFAs on skin conditions comes from in vitro studies, with limited data from clinical studies involving healthy volunteers or patient populations. This is a limitation for the scope of the findings reported herein since the effects observed in vitro are sometimes not reproducible in clinical trials, and the clinical application of the results from in vitro studies usually takes a long time. Since 2013, in the European Union, it is forbidden to sell cosmetic ingredients tested in animals [48]; hence, most of the industrial and academic research with cosmetic ingredients is limited to in vitro studies. Alternative skin models, based on human cells, reconstructed skin, ex vivo skin, organ‐on‐a‐chip, and computational in silico models, have emerged as a promising solution, as they are more cost‐effective and sustainable, present fewer ethical considerations, and, in certain cases, show advantages compared to the corresponding animal models [49]. As an example, we included in the manuscript some studies in which complex tissue culture models were used, such as the studies from Morin and Simard research group [24, 25, 27, 28, 29]. However, it should be emphasized that, despite its promising effects, the evidence of the topical effect of omega‐3 in humans is still weak.

It is worth noting that not only clinical studies in humans are needed to better understand the role of topical skin ω‐3 PUFAs, but also there are some experimental considerations that must be addressed, such as the most efficient way of delivering the ω‐3 PUFAs treatment to the skin. Although some studies assessed the bioavailability of ω‐3 PUFAs and their derivatives, showing their incorporation into cellular membranes, it is also known that DHA and EPA are highly susceptible to oxidative degradation [50]. This fact has led to the development of micro and nanoparticles to include the molecules and enhance their bioavailability, whereas other authors have opted to test directly the performance of other DHA derivatives [40].

Additionally, due to the inconsistency of treatments in composition, concentrations, excipients, and oil sources assessed, it is difficult to determine which is the best concentration, source, or composition of ω‐3 PUFA products for topical applications. Regarding the evidence from food supplements, ω‐3 microalgae oil seems to be a safer option for the skin than fish or krill oil, since there is a lower risk of content in contaminants, such as mercury or phthalates [51], which are harmful to skin cells [52]. Furthermore, algae are a more sustainable source of ω‐3 PUFAs than fish or krill, since microalgae harvesting in bioreactors reduces the risk of intensive fishing [51]. Consequently, the research of cosmetics ingredients based on ω‐3 PUFAs should be accompanied by the development of novel technologies for de novo production of ω‐3 PUFAs, such as microalgae or genetically engineered oilseed crops, as future sources of ω‐3 PUFAs [53, 54].

Finally, the effect of different molecular structures of ω‐3 PUFAs on the skin has not been previously evaluated, which contrasts with nutritional studies in which ethyl ester (EE), triglyceride (TG), re‐esterified triglyceride (rTG), free‐fatty acid (FFA), and phospholipid (PL) forms have been thoroughly studied. Most of the ω‐3 PUFAs are transformed, industrially, into EE to concentrate the fatty acids for food supplements [55]. Conversely, ω‐3 PUFAs are mostly found in TG and PL in natural matrixes, such as in marine products [56]. The EE structure allows for the manipulation of ω‐3 PUFAs and avoids the peroxidation and instability typically found in oils rich in FFA [57]; unfortunately, the EE oils have a lower bioavailability compared to TG or PL, as demonstrated in many studies [58, 59, 60, 61]. Consequently, with the main goal of commercializing ω‐3 PUFA supplements with enhanced bioavailability, there is a growing trend in the industry of ω‐3 PUFA supplements to re‐esterify oils rich in EE to reach more bioavailable structures such as TG and PL [62]. Since skin cells also possess lipases to absorb TG and PL, it could be assumed that TG and PLs will also be more bioavailable for skin cells, as happens in the digestive tract [63]. Therefore, it may be hypothesized that TG, PL, or re‐esterified TG would be better ω‐3 PUFAs structures for topical use as compared to EE or FFA.

## 5 Conclusion

This study provides a comprehensive review of the most recent evidence regarding the results of topical ω‐3 PUFAs for skin hydration and treatment of dermatological conditions. Overall, existing evidence suggests that ω‐3 PUFAs could be a promising option as an adjuvant or complementary treatment in managing several dermatological conditions. However, according to the evidence presented herein, there is a lack of consensus on the optimal dosage and delivery methods of ω‐3 PUFAs, and most of the studies are based on in vitro research, the application of which is far from clinical practice. Further preclinical and clinical studies involving healthy volunteers or patients are needed to elucidate the impact of the topical use of ω‐3 PUFAs and to determine the optimal delivery methods for these fatty acids in cosmetology and dermatology.

## Author Contributions

All authors provided the study concept and design. Ignasi Mora reviewed the literature, and all authors validated the data entry. Laura Mateu‐Arrom and Ignasi Mora wrote the initial draft. The final manuscript was seen and approved by all authors. The authors decline the use of artificial intelligence, language models, machine learning, or similar technologies to create content or assist with writing or editing of the manuscript.

## Ethics Statement

The authors have nothing to report.

## Conflicts of Interest

The authors declare no conflicts of interest.

## Supporting information


**Table S1.** Mammalian skin in vitro models treated with ω‐3 PUFAs (2018–2024).
**Table S2.** Preclinical mammalian skin models treated topically with ω‐3 PUFAs (2018–2024).
**Table S3.** Clinical trials with topical ω‐3 PUFAs treatments (2018–2024).

## Data Availability

Data supporting the findings of this review are available from the authors upon request.
