# Supplementary material for: Therapeutic Benefits of Topical Omega‐3 Polyunsaturated Fatty Acids in Skin Diseases and Cosmetics: An Updated Systematic Review
Source: J Cosmet Dermatol. 2025 Jul 4;24(7):e70341. doi: 10.1111/jocd.70341 (PMC12228025; doi:10.1111/jocd.70341)
Supplement: Supplementary file 1 — Table S1. Mammalian skin in vitro models treated with ω‐3 PUFAs (2018–2024). Table S2. Preclinical mammalian skin models treated topically with ω‐3 PUFAs (2018–2024). Table S3. Clinical trials with topical ω‐3 PUFAs treatments (2018–2024). [file JOCD-24-e70341-s001.docx]

**Therapeutic Benefits of Topical Omega-3 Polyunsaturated Fatty Acids in Skin Diseases and Cosmetics: An Updated Systematic Review**

**Laura Mateu-Arrom^1 (ORCID: 0000-0001-8522-4748)^, Ignasi Mora^2,3 (ORCID: 0000-0002-3861-8071)^ and Leia Garrote^4^**

*^1^Department of Dermatology, Hospital de la Santa Creu i Sant Pau, Institut d’Investigació Biomèdica Sant Pau (IIB Sant Pau), Universitat Autònoma de Barcelona (UAB), Barcelona, Spain*

*^2^Brudy Technology, S.L., Barcelona, Spain*

*^3^Universitat Rovira i Virgili, Tarragona, Spain*

*^4^Brudy Lab S.L., Barcelona, Spain*

Correspondence should be addressed to Ignasi Mora, Brudy Technology, C/ de la Riera de Sant Miquel, 3, 08006, Barcelona, Spain; e-mail: cultivos1@brudylab.com

| **Table S1.** Mammalian skin *in vitro* models treated with ω-3 PUFAs (2018-2024) | | | | |
| --- | --- | --- | --- | --- |
| **Treatment** | **Experimental Model** | **Target** | **Main Outcomes** | **Reference** |
| DHA and EPA at 10-30 μM in culture medium | B16F10 murine melanoma cells.  WM266-4 human melanoma cells | Melanoma | Sensitize tumoral cells to cisplatin through DUSP6/p-ERK/ERCC1 repair pathways. Cisplatin induces inhibition of cell growth and migration | Ottes-Vasconcelos *et al*. (2019) [1] |
| Resveratrol-stearate nanoparticles loaded with DHA at 10-30 μM | HaCaT and NCTC-2544 human immortalized keratinocytes | Skin irritation and inflammation | Inhibits production of inflammatory cytokines and ROS after induction with SDS and TNF-α | Serini *et al*. (2019) [2] |
| Chia seed extract (HYVIA™) with ALA and LA at 31.22 and 8.86 μg/mL respectively | Human epidermal keratinocytes (NHEKs) from neonatal donors | Skin hydration | Improves the expression of AQP3 and HAS2 skin-hydration markers | Huber *et al*. (2020) [3] |
| Treatment with 17-oxo-DHA or DHA at 1-20 μM | JB6 Cl 41 mouse epidermal cells | Stress response of skin cells | 17-oxo-DHA enhances the transcription factor Nrf2 and stimulates Keap1 degradation promoting autophagy | Jamil *et al*. (2020) [4] |
| Incubation for 24h with 20 µL of sacha inchi oil (composed by 42.3% of ALA) | Skin tissues females in the age range of 50‐65 years old after surgery | Skin damage after UV-B irradiation | Reduced secretion of TNF‐α and IL‐1. Reduced disruption of keratin 1 integrity in the stratum corneum layer | Soimee *et al.* (2020) [5] |
| Medium supplemented with 10 μM of DHA until the end of the culture | A 3D tissue culture with fibroblasts and keratinocytes from patients | Psoriasis | DHA decreases PGE_2_ and 12-HETE. Rebalances expression of PPARs and decreases TNFα. Overall, attenuates psoriatic characteristics | Morin *et al*. (2021) [6] |
| Culture medium with 10 μM of ALA | A 3D tissue culture with fibroblasts and keratinocytes from patients | Psoriasis | ALA increases EPA and ω-6 derivatives. Decreases psoriatic phenotype by normalizing keratinocyte proliferation and differentiation | Simard *et al*. (2021) [7] |
| Supplementation with 150 µM DHA or EPA | HaCaT human keratinocytes.  CRL-2522 newborn foreskin fibroblasts | Wound healing | Increases the metabolic activity of keratinocytes and fibroblasts. No significant positive effect on skin cell proliferation and wound closure | Severing *et al*. (2022) [8] |
| Culture medium supplemented with ALA | A 3D tissue culture with fibroblasts, keratinocyte and activated T cells from patients | Psoriasis | Reduces infiltration of T cells into the epidermis. Decreases inflammatory cytokines and chemokines (CXCL, IL-6 and IL-8) production | Morin *et al*. (2022) [9] |
| Oil with standardized levels of 17-HDHA, 18-HEPE, 14-HDHA, EPA, and DHA (LIPINOVA®). Dosage of 50 μM and 250 nM in cells | Human dermal fibroblasts and keratinocytes from patients. Monocytes (CD14+ cells) isolated from buffy coats of healthy donors | Wound healing and inflammation | Biocompatibility with keratinocytes and fibroblasts. Decreased expression of IL1β and CXCL10 genes, and lower amount of CXCL10 in macrophages. Promotion of *in vitro* wound closure | Ontoria-Oviedo et al. (2022) [10] |
| Culture medium with EPA 10 μM | A 3D tissue culture with fibroblasts, keratinocyte, and polarized T cells from patients | Psoriasis | Increases ω-3 PUFA in membrane phospholipids. Increases PGE_3_, 12-HEPE and EPEA levels. Regulates lipid composition of psoriatic skin and promotes homeostasis | Morin *et al*. (2023) [11] |
| Culture medium with DHA or ω-3 VLC-PUFA concentrate at 3 μM | PCS-201–012 human dermal fibroblast | Skin tissue development and integrity | Modulates gene expression and produce greater rate of fibroblast migration, favouring in-vitro scratch healing | Torrissen *et al*. (2023) [12] |
| Culture medium with 10 μM EPA | A 3D tissue culture with fibroblasts, keratinocyte, and polarized T cells from patients | Psoriasis | Normalizes the proliferation of psoriatic keratinocytes and modifies the NFκB pathway. Reduces the proportion of IL-17A-positive cells exerting anti-inflammatory effects | Morin *et al*. (2023) [13] |
| ALA: α-linolenic acid; DUSP6: dual- specificity phosphatase-6; ERK: extracellular signal-regulated kinase; ERCC1: protein excision repair cross-complementation group 1; SDS: sodium dodecyl sulfate; TNF-α: tumor necrosis factor alfa; AQP3: aquaporin 3; HAS2: hyaluronan synthase 2; 17-oxo-DHA: electrophilic α,β-unsaturated keto-derivative of DHA; PGE: prostaglandin E; 12-HETE: 12-hydroxyeicosatetraenoic acid; PPARs: peroxisome proliferator–activated receptors; CXCL: chemokine (C-X-C motif) ligand; IL: interleukin ; 12-HEPE: 12-hydroxyeicosapentaenoic acid; EPEA: N-eicosapentaenoyl-ethanolamine; VLC: very long chain; NFκB: nuclear factor kappa-light-chain-enhancer of activated B cells | | | | |

| **Table S2.**  Preclinical mammalian skin models treated topically with ω-3 PUFAs (2018-2024) | | | | |
| --- | --- | --- | --- | --- |
| **Treatment** | **Experimental Model** | **Target** | **Main Outcomes** | **Reference** |
| Dosage of 0.25, 0.5, 1, 2, and 4 mg of DHA (from fish oil) per ear | Healthy Wistar rats | Dermatitis by irritant substances | Successful penetration of fish oil in ear tissue. Inhibition of MPO content, indirect marker of neutrophil recruitment in inflamed tissue. No reduction of ear edema intensity | Ames *et al*. (2020) [14] |
| DHA (10 μmol), 17-oxo-DHA (20 nmol), and 17- OH-DHA (20 nmol) dissolved in 200 μl of acetone. Compounds applied onto the dorsal skin of mice for 2.5 or 5 h | Female HR-1, hairless mice | Stress response of skin cells | 17-oxo-DHA binds Keap1 and disrupt proteasome-mediated degradation of Nrf2. Elevates the nuclear localization of Nrf2 and expression of heme oxygenase-1 (HO-1). Overall, enhances antioxidant response | Jamil *et al*. (2020) [4] |
| Nanoemulsion containing fish oil or linseed oil as oil phase with tacrolimus (0.1% w/v), A single dose (500 mg) applied to the shaved skin of the mice (4 cm^2^ area) for one week | Adult albino mice (imiquimod induced psoriasis-like inflammation model) | Psoriasis | Treatment shows decreased PASI (Psoriasis Area and Severity Index) score and reduced inflammatory cytokine (TNF-α and IL-6) levels in the skin. Fish and linseed oil increase drug skin permeation and are promising carriers of tacrolimus for the treatment of psoriasis | Mittal *et al.* (2021) [15] |
| Daily dosage of 50 ng of LIPINOVA®, a triglyceride oil with standardized levels of 17-HDHA, 18-HEPE, 14-HDHA, EPA, and DHA | Adult type 2 diabetes (*db/db*) mice | Diabetic ulcers | Improves wound healing closure. Promotes thicker parakeratotic stratum corneum, mature dense connective tissue and induces angiogenesis. Macrophage phenotype switches from pro-inflammatory to pro-resolving | Ontoria-Oviedo *et al*. (2022) [10] |
| 17-Oxo-DHA (20 nmol) dissolved in 200 μL of acetone and applied in the dorsal skin of mice 30 min prior to UVB exposure | Female SKH1-Hr^hr^ mice (hairless) irradiated with UVB (180 mJ/cm^2^) | Dermatitis and skin cancer | Ameliorate cell death and reduce levels of oxidative stress markers: 4-hydroxynonenal (HNE)-modified protein, malondialdehyde (MDA), and 8-oxo-2′-deoxyguanosine (8-Oxo-dG). Increase the activation of Nrf2. Reduce TNFα and IL-6 production and expression. Reduce STAT3 phosphorylation (oncoprotein activator) induced by UVB. Protect against mouse skin tumour development | Kim *et al*. (2023) [16] |
| Emulsion-gel with 7 g of stingray liver oil per 100 g of product. The oil contains 14% of ω-3 PUFA. Aprox. 0.25 g of gel per rat’s paw (2.45 mg of ω-3 PUFAs) | Male Wistar rats, paw edema caused with intra-plantar injection of kaolin and dextran solution | Wound healing and edema | The treatment produced completely wound regeneration after 12 days and fast resolution of inflammatory edema after induction | Mititelu *et al*. (2023) [17] |
| Topical cream rich in PUFA extracted from microalga *Parachlorella kessleri* (ALA 22.76%) | Male Swiss albino mice (CD-1 strain) aged 6–8 weeks with two bilateral wounds or full thickness burns on the back | Burn and wound healing | Significant effect on the reduction of excisional wounds and burns. Histopathological analysis showed an improvement of angiogenesis, collagen fiber formation, and epidermis creation | El-Sheekh *et al.* (2024) [18] |
| ALA: α-linolenic acid; MCP-1: monocyte chemoattractant protein-1; MPO: myeloperoxidase; 17- OH-DHA: electrophilic α,β-unsaturated alcohol-derivative of DHA; Nrf2: nuclear factor erythroid 2-related factor 2; Keap1: kelch-like ECH-associated protein 1; HDHA: hydroxy-4Z,7Z,10Z,13Z,15E,19Z-docosahexaenoic acid; HEPE: hydroxy-5Z,8Z,11Z,14Z,16E-eicosapentaenoic acid; UVB: ultraviolet B; STAT3: signal transducer and activator of transcription 3 | | | | |

| **Table S3.**  Clinical trials with topical ω-3 PUFAs treatments (2018-2024) | | | | |
| --- | --- | --- | --- | --- |
| **Treatment** | **Patients** | **Target** | **Main Outcomes** | **Reference** |
| An emulsion formulated with 0.1% HYVIA™, chia seed extract with 31.22 μg/mL of ALA. Dosage of 50 µL applied in two 25 cm^2^ areas of the lower legs | 16 subjects, 5 males-11 females, age range from 18 to 60 | Skin moisturizing | Skin hydration was improved by 16% over vehicle at 2 hours post-treatment application. At 24 hours post-application, the treated skin was 58% more hydrated than the vehicle skin. The emulsion produced statistically significant lower transepidermal water loss values compared to untreated skin | Huber *et al*. (2020) [3] |
| Application in left or right lower leg of 0.5 mL of sacha inchi oil (composed by 42.3% of ALA) twice a day for 14 consecutive days | 13 healthy females in age range of 20‐60 years | Skin moisturizing efficiency and irritation potential | Beneficial effect on dry skin. Moisturizing effect comparable to that of olive oil. Proved safeness of the treatment | Soimee *et al.* (2020) [5] |
| A small amount (aprox. 0.2 mL) of gel-cream containing 0.7% of DHA in a triglyceride form in the skin of the right closed eyelids | 68 participants aged 22-60 years | Hydration and decongestion of eyelid skin | No reduction of inflammatory cytokine/chemokine in tears. Most of the participants reported improvements in skin brightness, softness, elasticity, and hydration, with no adverse effects | Pinazo-Duran *et al.* (2021) [19] |

**References**

1. Ottes Vasconcelos, R.; Serini, S.; De Souza Votto, A.P.; Santos Trindade, G.; Fanali, C.; Sgambato, A.; Calviello, G. Combination of ω-3 fatty acids and cisplatin as a potential alternative strategy for personalized therapy of metastatic melanoma: An in-vitro study. *Melanoma Res.* **2019**, *29*, 270–280, doi:10.1097/CMR.0000000000000564.

2. Serini, S.; Cassano, R.; Facchinetti, E.; Amendola, G.; Trombino, S.; Calviello, G. Anti-Irritant and Anti-Inflammatory Effects of DHA Encapsulated in Resveratrol-Based Solid Lipid Nanoparticles in Human Keratinocytes. *Nutrients* **2019**, *11*, 1400, doi:10.3390/NU11061400.

3. Huber, K.L.; Fernández, J.R.; Webb, C.; Rouzard, K.; Healy, J.; Tamura, M.; Voronkov, M.; Stock, J.B.; Stock, M.; Pérez, E. HYVIA^TM^: A novel, topical chia seed extract that improves skin hydration. *J. Cosmet. Dermatol.* **2020**, *19*, 2386–2393, doi:10.1111/JOCD.13469.

4. Jamil, M.U.; Kim, J.; Yum, H.W.; Kim, S.H.; Kim, S.J.; Kim, D.H.; Cho, N.C.; Na, H.K.; Surh, Y.J. 17-Oxo-docosahexaenoic acid induces Nrf2-mediated expression of heme oxygenase-1 in mouse skin in vivo and in cultured murine epidermal cells. *Arch. Biochem. Biophys.* **2020**, *679*, 108156, doi:10.1016/J.ABB.2019.108156.

5. Soimee, W.; Nakyai, W.; Charoensit, P.; Grandmottet, F.; Worasakwutiphong, S.; Phimnuan, P.; Viyoch, J. Evaluation of moisturizing and irritation potential of sacha inchi oil. *J. Cosmet. Dermatol.* **2020**, *19*, 915–924, doi:10.1111/JOCD.13099.

6. Morin, S.; Simard, M.; Flamand, N.; Pouliot, R. Biological action of docosahexaenoic acid in a 3D tissue-engineered psoriatic skin model: Focus on the PPAR signaling pathway. *Biochim. Biophys. Acta - Mol. Cell Biol. Lipids* **2021**, *1866*, 159032, doi:10.1016/J.BBALIP.2021.159032.

7. Simard, M.; Rioux, G.; Morin, S.; Martin, C.; Guérin, S.L.; Flamand, N.; Julien, P.; Fradette, J.; Pouliot, R. Investigation of Omega-3 Polyunsaturated Fatty Acid Biological Activity in a Tissue-Engineered Skin Model Involving Psoriatic Cells. *J. Invest. Dermatol.* **2021**, *141*, 2391-2401.e13, doi:10.1016/J.JID.2021.02.755.

8. Severing, A.L.; Rembe, J.D.; Füllerer, M.; Stürmer, E.K. Impact of the chronic wound microenvironment and marine omega-3 fatty acids on skin cell regeneration processes. *Exp. Dermatol.* **2022**, *31*, 725–735, doi:10.1111/EXD.14506.

9. Morin, S.; Simard, M.; Rioux, G.; Julien, P.; Pouliot, R. Alpha-Linolenic Acid Modulates T Cell Incorporation in a 3D Tissue-Engineered Psoriatic Skin Model. *Cells* **2022**, *11*, 1513, doi:10.3390/CELLS11091513/S1.

10. Ontoria-Oviedo, I.; Amaro-Prellezo, E.; Castellano, D.; Venegas-Venegas, E.; González-Santos, F.; Ruiz-Saurí, A.; Pelacho, B.; Prósper, F.; Pérez del Caz, M.D.; Sepúlveda, P. Topical Administration of a Marine Oil Rich in Pro-Resolving Lipid Mediators Accelerates Wound Healing in Diabetic db/db Mice through Angiogenesis and Macrophage Polarization. *Int. J. Mol. Sci.* **2022**, *23*, 9918, doi:10.3390/IJMS23179918.

11. Morin, S.; Tremblay, A.; Dumais, E.; Julien, P.; Flamand, N.; Pouliot, R. Eicosapentaenoic Acid Influences the Lipid Profile of an In Vitro Psoriatic Skin Model Produced with T Cells. *Biomolecules* **2023**, *13*, 1413, doi:10.3390/BIOM13091413/S1.

12. Torrissen, M.; Ytteborg, E.; Svensen, H.; Stoknes, I.; Nilsson, A.; Ostbye, T.K.; Berge, G.M.; Bou, M.; Ruyter, B. Investigation of the functions of n-3 very-long-chain PUFAs in skin using in vivo Atlantic salmon and in vitro human and fish skin models. *Br. J. Nutr.* **2023**, *130*, 1915–1931, doi:10.1017/S0007114523001150.

13. Morin, S.; Bélanger, S.; Ghio, S.C.; Pouliot, R. Eicosapentaenoic acid reduces the proportion of IL-17A–producing T cells in a 3D psoriatic skin model. *J. Lipid Res.* **2023**, *64*, 100428, doi:10.1016/J.JLR.2023.100428.

14. Ames, F.Q.; Bracht, L.; Sato, F.; Vizioli de Castro-Hoshino, L.; Ambrósio da Rocha, B.; Oliveira, L.A. de; Parreira de Lima, E.; Kenji Nakamura Cuman, R.; Luciano Baesso, M.; Aparecida Bersani-Amado, C. Fish oil preparation inhibits leukocyte recruitment and bands that characterize inflamed tissue in a model of phenol-induced skin inflammation: percutaneous penetration of a topically applied preparation demonstrated by photoacoustic spectroscopy. *Nat. Prod. Res.* **2020**, *34*, 2341–2345, doi:10.1080/14786419.2018.1533829.

15. Mittal, S.; Ali, J.; Baboota, S. Enhanced anti-psoriatic activity of tacrolimus loaded nanoemulsion gel via omega 3 - Fatty acid (EPA and DHA) rich oils-fish oil and linseed oil. *J. Drug Deliv. Sci. Technol.* **2021**, *63*, 102458, doi:10.1016/J.JDDST.2021.102458.

16. Kim, S.H.; Lee, S.E.; Kim, S.J.; Fang, X.; Hur, J.; Sozen, E.; Özer, N.K.; Kim, K.P.; Surh, Y.J. Protective effects of an electrophilic metabolite of docosahexaenoic acid on UVB-induced oxidative cell death, dermatitis, and carcinogenesis. *Redox Biol.* **2023**, *62*, doi:10.1016/J.REDOX.2023.102666.

17. Mititelu, M.; Licu, M.; Lupu, C.E.; Neacșu, S.M.; Olteanu, G.; Gabriela, S.; Drăgănescu, D.; Oancea, C.N.; Busnatu, Ștefan S.; Hîncu, L.; et al. Characterization of Some Dermato-Cosmetic Preparations with Marine Lipids from Black Sea Wild Stingray. *Mar. Drugs* **2023**, *21*, doi:10.3390/MD21070408.

18. El-Sheekh, M.; Bedaiwy, M.; Mansour, H.; El-shenody, R.A. Efficiency of the fatty acids extracted from the microalga Parachlorella kessleri in wound-healing. *Burns* **2024**, doi:10.1016/J.BURNS.2024.01.019.

19. Pinazo-Durán, M.D.; Raga-Cervera, J.; Sanz-González, S.M.; Salgado-Borges, J.; Benítez-del-Castillo, J.; Ramírez, A.I.; Zanón-Moreno, V. Efficacy and safety study of an eyelid gel after repeated nocturnal application in healthy contact lens users and non-users. *J. Optom.* **2021**, *14*, 28–36, doi:10.1016/J.OPTOM.2019.12.002.
